# No Early Effect of Intrathecal Rituximab in Progressive Multiple Sclerosis (EFFRITE Clinical Trial)

**DOI:** 10.1155/2021/8813498

**Published:** 2021-03-08

**Authors:** Mickael Bonnan, Sylvie Ferrari, Henri Courtade, Paul Money, Pauline Desblache, Bruno Barroso, Stéphane Debeugny

**Affiliations:** ^1^Service de Neurologie, Hôpital F. Mitterrand, 4 bd Hauterive, 64046 Pau, France; ^2^Pharmacie, Hôpital F. Mitterrand, 4 bd Hauterive, 64046 Pau, France; ^3^Biologie Médicale, Hôpital F. Mitterrand, 4 bd Hauterive, 64046 Pau, France; ^4^Service de Radiologie, Hôpital F. Mitterrand, 4 bd Hauterive, 64046 Pau, France; ^5^Département de Recherche Clinique, Hôpital F. Mitterrand, 4 bd Hauterive, 64046 Pau, France

## Abstract

**Background:**

The progressive phase of multiple sclerosis (MS) is characterized by an intrathecal (IT) compartmentalization of inflammation, involving B-cells within meningeal follicles, and resisting all the available immunosuppressive treatments. A new therapeutic paradigm may be to target this inflammation by injecting immunosuppressive drugs inside the central nervous system compartment.

**Methods:**

We designed a single-center, open-label, randomized, controlled, phase II study designed to evaluate the safety and efficacy of IT rituximab in progressive MS (EFFRITE trial; ClinicalTrial Registration NCT02545959). Patients were randomized into three arms (1 : 1 : 1): control group, IT rituximab (20 mg, IT) group, and intravenous+IT (IV+IT) group. The main outcome was a change in levels of CSF biomarkers of inflammation (osteopontin). Secondary outcomes were changes in levels of CSF biomarkers of axonal loss (neurofilament light chain) and clinical and MRI changes.

**Results:**

Ten patients were included (2 : 4 : 4). No adverse event occurred. OPN level remained stable in CSF at each time point, whereas NFL had slightly decreased (-8.7%) at day 21 (*p* = 0.02). Clinical parameters remained stable and leptomeningeal enhancements remained unchanged.

**Conclusion:**

Clinical outcome and biomarkers of inflammation were not dramatically modified after IT injection of rituximab, probably due to its limited efficiency in CSF. Drug issues for future studies are discussed.

## 1. Introduction

All the available treatments of multiple sclerosis (MS) target the relapsing phase of the disease, but no treatment is available to halt the secondary progressive (SP) phase, which remains the main cause of the evolving impairment associated with this disorder. The progressive phase is associated with persistent low-grade inflammation of the central nervous system (CNS), compartmentalized behind the blood-brain-barrier (BBB), and involving several processes including persistent oligoclonal bands and the presence of autoreactive T-cells and B-cells undergoing affinity maturation and somatic hypermutations (review in [1–3]). Extensive subpial demyelination is observed on the cortical ribbon in close association with minute ectopic lymphoid follicles, the tertiary lymphoid organs (TLO) [4]. Although a few of these structures could be observed on late gadolinium-enhanced FLAIR MRI sequences [5], which are described as leptomeningeal enhancing lesions (LME), many more tiny structures disseminated over the leptomeninges remain invisible to MRI. Rituximab may target the majority of CD20+ B-cells cuffing TLOs. Blood infusion of rituximab allows minimal diffusion within the CNS through the BBB (CSF : serum ratio 1 : 260), and its peak concentration in CSF remains very low (reviewed in [6]). Intrathecal (IT) infusion of rituximab overcomes the problem of diffusion in the CNS and was successfully and safely used to treat meningeal and brain B-cell lymphomas [7].

Drugs targeting CD20+ lymphocytes are highly efficient in preventing MS relapses but do not prevent SP MS impairment. The rationale of the study was to target the compartmentalized CNS inflammation, especially CD20+ lymphocytes of meningeal TLOs, by IT injection of rituximab in SP MS patients. Since rituximab is quickly cleared from the CNS due to the high level of CSF turnover, we focused our exploratory study on its short-term biological and clinical effects. Unfortunately, we obtained negative results as recently observed [8–11].

## 2. Study Design

### 2.1. Patients and Procedures

This single-center, open-label, randomized, controlled, phase II study called EFFRITE (Effet du rituximab intrathécal dans la SEP progressive) was designed to evaluate the safety and efficacy of IT rituximab in progressive MS. It was approved by the regional ethics board and the institutional research committee. All patients gave their written informed consent. Briefly, key eligibility criteria were primary or secondary progressive (PP or SP) MS for more than 2 years without clinical and radiological relapse, EDSS≥6.0, absence of immunosuppressive in the last 6 months, feasibility and tolerance of MRI and lumbar puncture (detailed criteria in Supplementary Table 1).

### 2.2. Sample Size

Patients were randomized (1 : 1 : 1) into three arms: control (Ctrl) group, IT rituximab (IT) group, and intravenous+IT (IV+IT) group. To avoid loss of opportunity, patients randomized to the control arm were allowed to rerandomize to the active arms after the completion of the study.

Rituximab infused into CSF is cleared into the blood flow within days and may also deplete extra-CNS B-cells, but the systemic effect of low-dose rituximab (20 mg) was unknown at the time of the study design. Therefore, we planned to offer IV rituximab to half of the IT-treated patients to ensure the complete depletion of blood B-cells and to prevent early reentrance in the CNS. This exploratory study was planned to enroll 12 patients.

### 2.3. Treatment and Follow-Up Protocol

Rituximab dose was 20 mg IT (diluted to 10 mL in isotonic solution) and 375 mg/m^2^ IV given once. IT rituximab was gently infused for 1 min under strict asepsis following a depletive sampling (10 mL) lumbar puncture (LP) obtained by gentle suction with a 5 mL syringe. The patient was in the Trendelenburg position (15°) for one hour to maximize diffusion of lumbar CSF to the brain. All patients including controls received pretreatment drugs (paracetamol 1000 mg, dexchlorpheniramine 5 mg, and methylprednisolone 120 mg IV) given to prevent clinical manifestations of allergy and cytokine release syndrome. IV rituximab was started one hour after IT injection.

**Figure 1:**
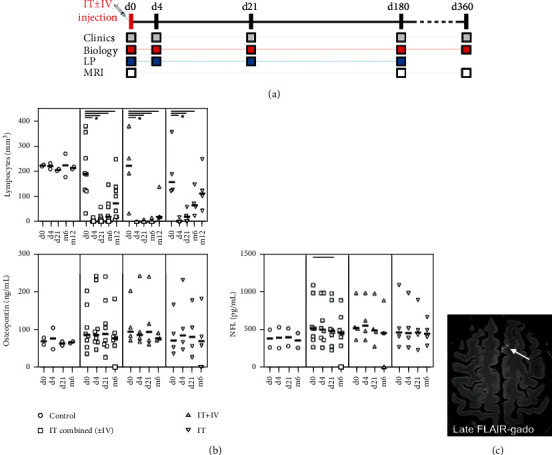
Study timeline and main results. (a) Schedule of investigations (from day 0 to 360). (b) Lymphocyte count decreased in treated groups. OPN and NFL levels remained mostly stable over time. ^∗^*p* < 0.05. (c) Leptomeningeal enhancing lesion (LME) on basal MRI (#6), late-acquired axial FLAIR-enhanced sequence. Absence of overt change in control MRIs (not shown).

A CSF aliquot (2 mL) was used for biochemistry and cell count, the other was put on ice and centrifuged, and the supernatant was frozen (-80°C) until biomarker tests. Osteopontin (OPN) and neurofilament light chain (NFL) were measured using ELISA kits (RD System, dilution 1 : 100; IBL, dilution 1 : 2). Clinical examination and blood sampling were scheduled at day 0, day 4, day 21, and months 6 and 12. CSF was sampled at each visit up to month 6 (Figure 1(a)).

### 2.4. Outcome Assessments

The primary objective was the evolution of OPN in CSF, which is a marker of CNS inflammation. Secondary outcome was the evolution of NFL level in CSF, which is a marker of neurodegeneration. Other endpoints were safety, clinical benefits, IgG synthesis (OCB, IgG index, IgG_Loc_), and radiological changes.

Clinical endpoints were Expanded Disability Status Score (EDSS), Timed 25-foot walk (T25FW), Symbol Digit Modalities Test (SDMT), Nine Holes Peg Test (NHPT), Modified Fatigue Impact Scale (MFIS), analogic fatigue scale (AFS), and clinical examination. Maximal score (300 sec) was assigned to aborted assays of NHPT. Subjective impressions of patients were recorded. Clinical ratings were done by unblinded investigators (MB, BB).

Brain MRI scans (3 T-magnet, Phillips) were performed before treatment/LP at day 0, month 6, and month 12. MRI protocols included conventional sequences (FLAIR), volumetric T1 sequences (echo time/repetition time = 3.1 ms/6.7 ms, flip angle 8°, 1.2 mm slice, with no gap, in-plane resolution 1mm^2^, FOV 256 × 256 mm^2^) acquired before and after gadolinium injection, and late acquired (≥20 min) contrast-enhanced 3D-FLAIR sequences.

### 2.5. Statistical Analysis

Data are given as median [min-max] or mean ± SD. The Mann–Whitney-Wilcoxon (MWW) rank-sum test and the Kruskal-Wallis (KW) test by rank were used to compare biological values between two and three groups, respectively. To avoid biases due to the limited size of the control group, we analyzed changes from baseline biological values by two different approaches. The distribution of the difference of each time point value with mean basal values was compared by the MWW test (between two groups) and by the KW test (between three groups). In a second approach avoiding the control group and pooling treated groups, the distribution of values at each time point was compared with baseline values using the Wilcoxon signed rank test (WSR).

Clinical data were recorded as improved (+1), stable (0), or worsened (-1) by comparison with baseline values, according to predefined cut-offs of meaningful changes from baseline exceeding: ≥0.5 point in EDSS; ≥20% in T25FW and NHPT; ≥10% in SDMT, MFIS, AFS. Results from the pooled treated group were compared with the control group by the Fisher bilateral test (FBT), and changes from baseline over time among the pooled treated group with the z-test for proportions. The SAS package was used for statistical analysis, and figures were plotted with GraphPad Prism 8.0.0 for OsX (GraphPad San Diego, http://www.graphpad.com).

## 3. Results

### 3.1. Study Population

All screened patients were included, and all of them completed the follow-up. Data were derived from 10 inclusions (Controls: 2, IV: 4, and IV+IT: 4) (Table 1). Due to inclusion difficulties and to negative results from other earlier studies, enrollment was interrupted although only two control patients were included. Both were initially randomized to the control group but were subsequently rerandomized to an active arm. Median age at inclusion was 62 years (49 to 68), 2 were PP-MS, and 6 were SP-MS. The median EDSS at baseline was 6.5 [6 to 8]. The median duration of MS was 20 years (6 to 42). The first patient was treated in November 2015, and the final collection of data occurred in May 2019.

### 3.2. Blood B-Cell Depletion

Basal mean CD19+ B-cell lymphocyte count was normal in each group (198-223/mm^3^), and rituximab infusion induced dramatic B-cell depletion as soon as day 4, followed by differential recovery among the groups (Figure 1). Complete B-cell depletion was achieved at day 4 after high-dose rituximab (IV+IT group). It was sustained for 6 months (4/mm^3^ [0-16]), but recovery was still incomplete at month 12 (46/mm^3^ [7 to 138]). In the IT group on low-dose rituximab, complete B-cell depletion was achieved in 3 of 4 patients (group mean 4/mm^3^ [0 to 17]) at day 4, but recovery started as soon as day 21 (24/mm^3^ [0 to 58]) and progressed over a year to incomplete values (128/mm^3^ [43 to 248]).

### 3.3. CSF Changes

Median CSF volume sampled was 10 mL (7 to 12 mL). Cell count, protein level, and glucose were in the normal range, and red cell count remained low (<5 red cells/mL). A minimal decrease in Q_Alb_ (BBB permeability) was observed in the pooled treated group at day 4 (5.37 × 10^−3^, -18.3%; *p* = 0.055) and at day 21 (-8.8%; *p* = 0.11), as expected after steroid infusion. IgG index and OCB remained stable in all cases. IgG_Loc_ was slightly increased in the pooled treated group at day 4 (10.9 g/L, +46%; *p* = 0.01), suggesting a direct but very transient consequence of restricted BBB permeability. However, values at day 21 and month 6 remained close to baseline. No statistical difference was observed between the treated and control groups for Q_Alb_ and IgG_Loc_ values.

No significant differences in median CSF level of NFL and OPN were observed between the three groups (KW) or using pooled treated groups (MWW). Changes in CSF level of NFL and OPN over time were not different between controls and pooled (MWW) or nonpooled (KW) treated groups. Unfortunately, basal CSF levels of OPN (68.6 ± 13.1 ng/mL vs. 100.5 ± 56.7 ng/mL) and NFL (377.6 ± 167.4 pg/mL vs. 581.5 ± 295.7 pg/mL) were lower in control than in treated patients, so a definitive group comparison was not possible. Furthermore, the control group was undersampled.

To avoid this bias, we analyzed changes over time by intragroup comparison in the pooled treated group at each time point versus baseline (WSR). OPN level remained stable at each point. NFL level was stable at day 4 (*p* = 0.38) and had decreased slightly at day 21 (-8.7%; *p* = 0.02) and at day 180 (*p* = 0.09), although the decrease was not significant.

### 3.4. Clinical Outcome

None of the patients experienced MS relapse during the follow-up period. EDSS remained unchanged. Fatigue scores (MFIS and AFS) and SDMT remained roughly stable. Ambulation was already lost at baseline in 3 patients (IT: 2, IV+IT: 1), so they did not undergo T25FW. No improvement was observed in pooled results from the active groups. Severe limitation of hand movements was observed in 3 patients, and assays were aborted in 11 examinations (4.5%). The median time to perform 9HPT remained unchanged in each group. Results remained unchanged even when data from the paretic arm were discarded. No difference in clinical scores was observed between control and treated patients (FBT). Considering pooled treated patients, a trend to improvement (z-test) was observed at day 4 (changes towards improvement: 68%; *p* = 0.07) and day 21 (66.7%; *p* = 0.10), but it had disappeared at later time points.

### 3.5. Adverse Events

IT injection of rituximab and the Trendelenburg position were well tolerated, and no adverse event was observed in relation with injection. All LP procedures could be performed, except one which failed after multiple attempts (M6 in IV+IT group).

### 3.6. MRI Changes

Lesion load remained unchanged on follow-up imaging, without any new white matter lesion or enhancement on T1w sequences. At least one LME was observed in 5 of the treated patients on baseline MRI (Figure 1(c)). No new LME appeared and enhancement intensity of baseline lesions remained stable at follow-up (days 180 and 360).

## 4. Discussion

### 4.1. Discussion of Results

Since no treatment is available for progressive MS, drugs crossing the BBB to safely target persisting IT low-grade inflammation are eagerly awaited. Rituximab was initially a promising candidate for three reasons: its efficacy on extra-CNS inflammation, its exclusion from the CNS by BBB after blood injection preventing action within the CNS, and its safety profile, which has already been demonstrated for IT use in CNS lymphomas. Our study and others confirm that the safety profile of the IT injection of rituximab makes it a candidate for use in MS patients. We observed none of the side-effects (headache, back pain, painful paresthesia of the buttocks, and chills) classically observed in association with injection of higher dosages, since they were probably related to tumor lysis syndrome in the context of high lesion load in IT lymphomas but not in MS, and they were prevented by systematic pretreatment (reviewed in [6]).

The positive effect of a treatment halting progressive MS would be difficult to capture by clinical examination only. Indeed, and as already observed, no meaningful change was observed on clinical parameters. The pathophysiology of fatigue, which is one of the major causes of clinical impairment in MS, is poorly understood but may involve bathing the CNS with high levels of CSF cytokines. Since one of the objectives of the study was to strongly reduce CNS and CSF inflammation, we expected to detect an early transient effect on fatigue. Unfortunately, we did not observe any such effect. All clinical parameters remained stable throughout the follow-up and failed to capture any beneficial effect, as observed previously with other treatments [12]. These disappointing results are in line with those previously observed. While SDMT score was minimally improved in one study [9], no change was observed in any other parameters [8–11].

We focused on early changes potentially induced by IT injection based on known pharmacokinetic data. Unlike blood-infused rituximab, the drug is cleared from the CSF within a few days [10, 13, 14], so long-lasting B-cell depletive activity is prevented in the CSF compartment. Even if intrathecally injected rituximab were to be very efficient, the probability of depleting all CNS B-cells after a single injection would be low. We therefore think that the maximal effect would be reached during the first days after lumbar infusion, but that it would quickly wane due to repopulation of the CNS compartment from blood-borne or spared CNS-resident B-cells. We therefore sampled CSF both very early (day 4) and later in order to capture any transient effect of the drug, but no significant early changes were evidenced.

Ideally, the objective would be to suppress the CNS inflammation underlying progressive MS. Since progressive MS is slowly degenerative, CSF biomarkers are able to predict late evolution and could be early markers of drug effects. To investigate persistent inflammation, we focused on OPN, which is a pleiotropic sensitive biomarker of CNS inflammation that is synthesized by most immune cells and is involved in the differentiation of Th-17 T-cells. Levels of OPN are higher in the CSF of MS patients than in healthy controls or other inflammatory disorders [15–19] and are sensitive to the effect of drugs in progressive MS [20]. Unfortunately, however, no major change in CSF OPN was observed at any time point.

Changes in degenerative profile were assessed by CSF NFL levels, which are predictive of 10-year outcome, mirror the rate of CNS atrophy, and could be a future predictive biomarker of outcome [21]. They are always high in progressive or uncontrolled MS patients, while they decrease in drug-responding patients. As in previous studies, no dramatic change in NFL levels was observed in our patients. Contrary to the initial high expectations, no clear-cut effect on any biological CSF parameter has been obtained in any IT rituximab trials [8–11] (Table 2), although low-dose rituximab was unexpectedly efficient in depleting blood B-cells.

At least part of the pathophysiology of progressive MS is due to cortical and meningeal inflammation (TLO) [3, 22]. This process is partly and indirectly captured by MRI, late-acquired gadolinium-enhanced FLAIR sequences showing foci of leptomeningeal enhancements (LMEs), whereas subpial cortical lesions are too faint for meaningful interpretation. Although TLO are mainly composed of CD20+ B-cells, we and others failed to observe any change in the aspect of LME after treatment [11]. Resistance to IT rituximab could be due to several causes: disconnection between B-cell targeting and LME, failure to reach these structures, or B-cell resistance to rituximab nested in survival niches. Future studies in animal models could help to explore these hypotheses.

The spatial relationship between TLO and subpial lesions may be more complex than the initially supposed local bystander toxic effect. The fate of these tiny structures remains unknown, and only some of them tend to vanish over time [5] (unpublished observations), possibly obscuring the spatial relation between LMEs and subpial lesions. Studies using 7 T-MRI imaging failed to correlate LME with focal subpial lesions but did replicate the known association between LMEs and cortex thinning [23], suggesting that LMEs may constitute or be part of a nonlocal process. However, the very high spatial resolution obtained with 7 T-MRI from the extension of subpial lesions is less informative than pathological exam, and LME count remains largely underestimated by 7 T-MRI. For example, LMEs were observed in 80% of SP-MS and the mean number of LMEs was 3.2 ± 3.3 using 7 T-MRI [23], whereas pathological exams revealed a mean of 6 ± 3 TLO per brain block, or up to 18% incidence per 4cm^2^ brain area. In EAE models, careful examination of whole brain revealed up to 60 TLO per rat brain [24, 25]. Therefore, although it is appealing to select patients to receive IT drugs on the basis of LME(+) criteria, the presence of LMEs seems an insufficient and probably too selective criterion.

Although trials of IT rituximab failed to demonstrate any substantial effect on clinical, biological, and MRI outcomes, there are clues to the causes of failure that may help in designing future studies. Very low dosages of rituximab (1 to 25 mg) injected in CSF (but rapidly cleared to blood) were able to completely deplete B-cells from blood [8, 10, 11, 26]. This strong effect of low-dose rituximab outside the CNS compartment suggests that the IT dosage was sufficient to deplete the CNS compartment of B-cells and confirms that the conditions required for drug efficiency in CSF, which are reached in CNS lymphomas, are not scalable to MS patients. Although CSF B-cells were depleted after IT rituximab, they were still detectable and rose in a few weeks, long before observable changes in blood B-cell count occurred [8, 11, 26]. These results were reproduced both after IT and ventricular infusions, suggesting that inadequate dispersion of rituximab in CSF was probably not the main limiting factor.

Soluble CD21 (sCD21), which is a marker of B-cell pool, was decreased in serum after rituximab infusion in relation with systemic B-cell depletion, whereas CSF sCD21 was less involved [8, 11]. These results are supportive of B-cell depletion mainly restricted to CSF-floating cells but sparing CNS-resident B-cells, which could a possible consequence of the limited diffusion of rituximab into brain tissue.

### 4.2. Study Limitations

This study has several limitations. First, since the safety of the procedure had not yet been assessed when this phase 2 trial was initiated, we planned to include a limited number of patients. However, we were unable to include all the expected patients due to the invasiveness of the procedure and because other treatments were being offered elsewhere at this time. Second, there was an inclusion bias towards severe and older patients. The lack of clinical and radiological activity during the study period was not considered to be a limitation, since the main goal of the study was dedicated to CSF biomarkers. Third, while we focused exploratory biological tests on highly sensitive markers of inflammation and neurodegeneration, we cannot rule out that a transient biological effect may have been missed and that it might have been revealed by other markers. Investigations were limited to the main biomarkers available in our center, and CSF B-cell count was unavailable. We cannot exclude that methods with improved sensibility to test OPN and NFL (e.g., SIMOA method) may have allowed the detection of small differences induced by treatment. Lastly, drugs were administered by lumbar puncture so we cannot be sure that they diffused homogeneously to higher levels, although diffusion is widely thought to be optimal. Moreover, it was recently demonstrated that rituximab activity was unexpectedly and dramatically decreased in the CSF bath, in relation with the unavailability of effectors. Concentrations of CD56^dim^ NK-cells, which are involved in ADCC, are far lower in CSF (<0.1%) than in blood [27–29]. The concentration of complement in CSF is less than 1% of serum concentration [30, 31], whereas its level is increased in CNS lymphomas and in various CNS disorders [13]. Therefore, IT rituximab was associated with a lower decrease in CSF B-cells than in serum (-80% vs. -98%), and changes in the indirect biomarkers of CSF B-cell activity (sCD21, BAFF) remained moderate [10, 11, 29].

Finally, this work and others failed to efficiently target CNS B-cells [8–11], and alternative strategies should be envisioned to reduce leptomeningeal inflammation. As an example, Bruton's tyrosine kinase (BTK) inhibitors, which target B-cell and myeloid cell activation, and are able to cross the BBB, demonstrated an effect in a mice model [32]. Phase 2 trial of oral evobrutinib in relapsing-remitting MS showed a reduced relapse rate [33], and future studies could examine the effects of BTK inhibition on leptomeningeal inflammation in progressive MS.

## 5. Conclusion

Although biological or clinical changes obtained after IT treatment were minor, its rationale remains well-founded although the methodology should be completely redesigned. The key factor prompting the choice of rituximab was its widespread safe IT used to treat lymphomas, without precluding the fact that similar conditions of efficiency could be reached in MS patients. Since B-cells are poorly targeted by IT rituximab, the absence of impact on the biological markers of neurodegeneration does not rule out IT B-cells being valuable targets in the future, but care must be taken to choose the appropriate drugs that are active in CSF.

## Figures and Tables

**Table 1:** Patient characteristics. Patients 9 and 10 were, respectively, patients 7 and 8 rerandomized to active groups after completion of study in control arm. CP: cyclophosphamide; Ctl: control group; DMF: dimethylfumarate; IFN: interferons; IT: intrathecal rituximab group; IT+IV: intrathecal and intravenous rituximab group; MTX: methotrexate; PP: primary progressive MS; SP: secondary progressive MS.

#	Group	Gender/age	MS type	Disease duration (years)	Baseline EDSS	Previous therapies
7	Ctl	F/49	SP	16	6.5	CP, DMF
8	Ctl	F/67	SP	40	6	None
1	IT	M/64	PP	15	6	MTX
4	IT	M/50	SP	25	7	IFN, CP
5	IT	F/65	SP	35	8	MTX
6	IT	M/60	SP	6	6	MTX, CP
2	IT+IV	F/62	PP	16	8	MTX, IFN
3	IT+IV	M/61	SP	22	7.5	Steroids
9	IT+IV	F/52	SP	18	6.5	CP, DMF
10	IT+IV	F/68	SP	42	6	None

**Table 2:** Studies of intrathecal rituximab in progressive MS.

Ref.	(*n*) ratio^a^	EDSS baseline	Clinical/MRI activity (≤1year)	Rituximab protocol	Biomarkers
[8]	23 1.5 : 1	6.5 2.5-7.0	Yes	IT LP 25 mg ×2 IV 200 mg ×2	IgG index, NFL, CXCL13, CCL19, sCD14: unchanged; IL12p40 -42%, BAFF +8%, sCD21, sCD27: minimal changes.
[9]	23 1 : 0	6.5 4.0-7.5	No	IT Om. 25 mg ×3	NFL, GFAP, MBP, Gal-9, sCD27, CXCL14: unchanged; CXCL13: minimal decrease.
[10]	9 1 : 0	5.5 4.0-8.0	Yes	IT LP 5-10-15 mg	*λ* FLC, CXCL13: unchanged; *κ*FLC: minimal increase; BAFF: decreased
[11]	8 1 : 0	6.0	No	IT LP 25 mg ×2	NFL, sCD21, sCD27, sCD14, CCL19, CCL21: unchanged; BAFF increased; CXCL13: decreased.
Our	10 4 : 1	6.5 6.0-8.0	No	IT LP 20 mg ±IV 375 mg/m^2^ (1 : 1)	IgG index, OCB, NFL, OPN: unchanged.

IT: intrathecal; LP: lumbar puncture; Om.: Ommaya reservoir. ^a^ratio active : control.

## Data Availability

Raw data can be obtained from corresponding author upon reasonable request.

## Additional Points

*Highlights.* Inflammation within intrathecal compartment is characteristic of progressive MS. Intrathecal injection of drug overcomes exclusion by blood-brain barrier. Intrathecal rituximab was safe but failed to change CSF biomarkers. Rituximab is not effective in CSF, so other drugs should be preferred.

## Supplementary Materials

Supplementary Materials Supplementary Table 1: inclusion and exclusion criteria. Supplementary Table 2: CONSORT 2010 flow chart.
