# Supplementary material for: No Early Effect of Intrathecal Rituximab in Progressive Multiple Sclerosis (EFFRITE Clinical Trial)
Source: Mult Scler Int. 2021 Mar 8;2021:8813498. doi: 10.1155/2021/8813498 (PMC7964121; doi:10.1155/2021/8813498)
Supplement: Supplementary Materials — Supplementary Table 1: inclusion and exclusion criteria. Supplementary Table 2: CONSORT 2010 flow chart. [file 8813498.f1.docx]

| **INCLUSION CRITERIA** |
| --- |
| Age ≥45 years, male or female  Primary or secondary progressive MS ≥2 years  EDSS ≥6.0  Absence of immunosuppressive drugs ≥6 months  Absence of licensed treatment of progressive MS  Absence of contra-indication to Trendelenburg position  Compliance with protocol and exams  Absence of parenchymal contrast uptake on T1 sequence at inclusion, except meningeal enhancement on late acquired FLAIR-enhanced sequence  Absence of pregnancy, menopause or active contraception during study  Social insurance coverage  Absence of concomitant inclusion in research protocol  Cognitive preservation sufficient to understand protocol and consent to medical procedures |
| **EXCLUSION CRITERIA** |
| Recurrent-relapsing (RR) MS  Documented MRI relapses during preceding 2 years  Contra-indication to lumbar puncture (local lesion, risk of hemorrhage, anticoagulant or antiplatelet drugs) or expected technical problems  Contra-indication of MRI  Vulnerability to infections  Severe cardiac insufficiency  Immunosuppressive drug or steroids during last 6 months  Autoimmune disorders (other than MS) requiring or susceptible to require immunosuppressive drug  Previous use of anti-CD20 drugs  Dementia or any severe psychiatric condition limiting ability to provide informed consent |

**Supplementary table 1. Inclusion and exclusion criteria**

**Supplementary Table 2. CONSORT 2010 flow diagram**

Allocated to intervention arms (n=8)

♦ Received allocated intervention (n=8)

including IT alone (n=4) or IT+IV (n=4)

Analyzed (n=2)
♦ Excluded from analysis (n=0)

Analyzed (n=8)
♦ Excluded from analysis (n=0)

## Analysis

Lost to follow-up (give reasons) (n=0)

Discontinued intervention (give reasons) (n=0)

Lost to follow-up (n=0)

Discontinued intervention (n=0)

## Follow-Up

Allocated to control arm (n=2)

♦ Received allocated intervention (n=2)

Excluded (n=2)

♦  Declined to participate (n=2)

## Enrolment

## Allocation

Randomized (n=10)

Assessed for eligibility (n=12)
